# The Relationship between Social Mentality and Health in Promoting Well-Being and Sustainable City

**DOI:** 10.3390/ijerph191811529

**Published:** 2022-09-13

**Authors:** Zhen Liu, Guizhong Han, Jiajun Yan, Zhichao Liu, Mohamed Osmani

**Affiliations:** 1 School of Design, South China University of Technology, Guangzhou 510006, China; 2 Management School, Guangzhou City University of Technology, Guangzhou 510800, China; 3 School of Architecture, Building and Civil Engineering, Loughborough University, Loughborough LE11 3TU, UK

**Keywords:** social mentality, mental health, COVID-19, comparative study, bibliometric, sustainable development, youth, city

## Abstract

In the context of the coronavirus disease 2019 (COVID-19), people’s social mentality and mental health have been severely affected, which has hindered or even reversed the achievement of the United Nations Sustainable Development Goals (SDGs). However, there is a lack of investigation into the potential relationship between social mentality and health, as well as of the comparison between different databases worldwide and in China, in the current context of COVID-19. Hence, the aim of this paper is to explore the research hotspots and development trends of social mentality and health in China and worldwide, while improving people’s health, building a sustainable society, and facilitating the achieving of the SDGs. A bibliometric method is employed in this paper from a macro-quantitative and micro-qualitative perspective to explore the research hotspots and trends of social mentality and health in the world and China from the two databases, namely the English-language Web of Science (WOS) and the Chinese-language China National Knowledge Infrastructure (CNKI). The results indicate that: (1) By using keyword co-occurrence and clustering analysis via the CiteSpace software bibliometric tool, 11 current research hotspots have been identified and studies are increasing in terms of using the Chinese language and the English language. (2) The current studies in the CNKI database mainly focus on the macro social environmental factors affecting social mentality and population research, while the studies in the WOS database pay more attention to social mentality and health in the context of the COVID-19 epidemic situation and a variety of professions. Hence, future research could explore the influencing factors and cultivation methods toward a healthy social mentality from the perspective of methodology and toward achieving SDG 3, providing healthy lives and promote well-being for all at all ages, and SDG 11, building sustainable cities and communities in the post-pandemic COVID-19 era.

## 1. Introduction

At present, the coronavirus disease 2019 (COVID-19) has already affected mental health worldwide and a number of effects are likely to persist [1], such as anxiety, depression, and post-traumatic symptoms [2]. This has the potential to reverse progress toward United Nations Sustainable Development Goal (SDG) 3, which aims to ensure healthy lives and well-being for all [3]. However, sustainable mental health at the individual level can form a healthy social mentality [4] and the public’s attitude toward COVID-19 reflects a nation’s culture, quality, and competitiveness, in which a healthy social mentality is conducive to dealing with the current and post-COVID-19 situations [5]. Harmonious societies can be achieved and supported by a healthy social mentality [6], which facilitates sustainable cities and communities in line with the United Nations SDG 11 [7].

In addition, sustainability is one of the most important goals on the world policy agenda [8]. The city is one of the key drivers of sustainable development to which has been applied the concept of the so-called sustainable city [9]. A sustainable city is where achievements in social, economic, and material development can be sustained [10], and in which the social index is one of the important indicators in sustainable city development [11]. It can be seen that a sustainable society is an important part of achieving a sustainable city, whilst a sustainable society is a society that ensures the health and vitality of human life and the culture and natural capital for present and future generations [12]. In the process of measuring sustainability, a sustainable society is defined as a society in which people are able to develop themselves in a healthy way [13], while the health of people (i.e., physical, mental, and emotional) is not affected by structural barriers in sustainable societies [14]. Thus, individual well-being is the foundation of strong and sustainable societies [15]. Achieving sustainable development worldwide requires a fair and balanced social environment [16], where scientific achievements in all fields are also essential to ensure sustainable societies [17]. COVID-19 has changed the world from what it used to be, but it also offers the opportunity to build better and more sustainable societies and cities [18]. Moreover, the need of people for a better society is the basis and driving force for the formation of a healthy social mentality [19].

Social mentality is the social state of mind in the whole society or in social groups in a certain period of social development, which is also the sum of the feelings, social emotional tone, social consensus, and social values of the whole society [20]. Social mentality has been defined in three ways from the perspective of philosophy and the relationship between social form and social psychology [21]. As one of the most general and complex social psychology phenomena, the formation mechanism of social mentality is extremely complex [22], with a system composed of three levels of psychological orientation, social reason, and spiritual support and its various elements [23]. It is also comprehensively influenced by various factors of social existence, social consciousness, and social subjects (i.e., social groups and individuals) [24]. Meanwhile, the current research on social mentality is mainly focused on the macro environment analysis of social change [25] and the human-centered micro environment and other factors affecting social mentality [26], as well as human mental-health-based interpersonal relationships and social mentality [27]. Therefore, social mentality has the characteristics of sociality, class, nationality, and times [28]. Although these are widely cited and accepted concepts of social mentality, it is an ambiguous concept in China’s social science research [29]. Additionally, although the current research on social mentality is more and more in-depth, a fundamental theoretical system of social mentality has not been formed [30].

Furthermore, current research believes that social mentality has both positive and negative effects on progress of social development, and a great significance to social stability [31]. A positive social mentality can promote social cohesion, innovation levels, and social governance [32], but social emotion has also changed after the outbreak of COVID-19 and it will require a long time to repair mental health, social emotion, and social mentality [33]. Therefore, Zhao et al. [34] believed that the relationship between mental health and social mentality, especially the correlation and intervention measures of healthy social mentality, need further research. However, there is still a lack of comparative research on social mentality and health worldwide, especially in the current context of COVID-19. For example, the compared studies of the theory and practice of social mentality in the world and China can be a collected experience and be reflected on for future research [35]. Hence, this paper aims to analyze the research on social mentality and health in China and worldwide and explore the hotspots and development trends of social mentality and health research, so that researchers can understand the research status and provide them with potential research directions in the future, and finally achieve SDGs 3 and 11, especially in the context of COVID-19.

## 2. Methods

A bibliometric method has been adopted for this study to explore the research hotspots and development trends of social mentality and the potential relationship between social mentality and health from macro quantitative and micro qualitative perspectives via two knowledge databases, namely the China National Knowledge Infrastructure (CNKI) [36] and the Web of Science (WOS) [37]. The bibliometric method is a research method that combines information science with mathematics and statistics [38]. The CNKI is the largest and key Chinese-language literature database in China, covering more than 99% of Chinese academic and practical journals [39], and the WOS is worldwide one of the largest and most used English-language databases and an appropriate database when it comes to performing multidisciplinary and international bibliometric analysis [40]. Bibliometric analysis is a quantitative analysis method that takes various external characteristics of the scientific and technological literature as the source of research, and uses mathematical and statistical approaches to describe, evaluate, and predict the current situation and development trend of the research [41]. Hence, CiteSpace 5.8. R3 software has been employed as the bibliometric tool. The CiteSpace software is an information visualization software developed by Dr. Chaomei Chen from Drexel University, which is mainly used for the measurement and analysis of scientific literature data [42]. It can take the title, research institution, core author, and keywords of the studied literature as the main analysis objects, and present the structure in the form of visualization based on the pathfinding network algorithm theory and co-citation analysis approach [43]. It provides a platform for visual analysis of the dynamic evolution process of the knowledge domain [44].

**Figure 1:**
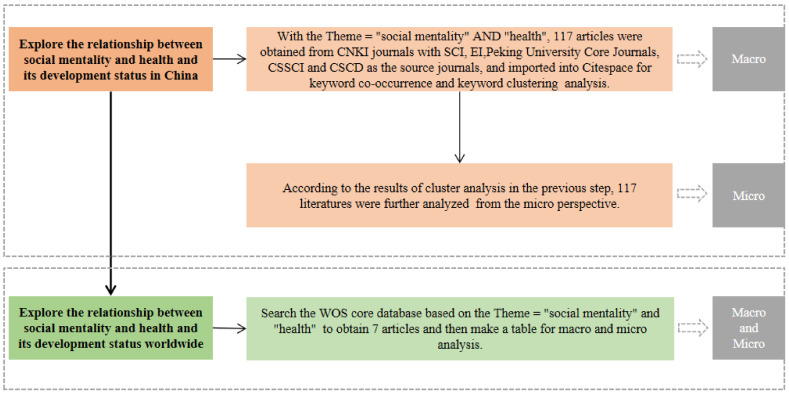
Research flow chart (generated by authors).

As shown in Figure 1, the study has been conducted as follows: we (1) explored the relationship between social mentality and health and its development status in China. With the themes “social mentality” AND “health”, 117 articles were selected from the CNKI database (accessed on 1 April 2022), with the Science Citation Index (SCI), the Engineering Index (EI), the Peking University Core Journals, the Chinese Social Sciences Citation Index (CSSCI), and the Chinese Science Citation Database (CSCD) as the source journals, and imported into the CiteSpace software for keyword co-occurrence and keyword clustering analysis from the macro perspective; (2) conducted further micro analysis on the 117 articles based on the clustering in step 1; and (3) explored the relationship between social mentality and health and its development status worldwide. With the themes “social mentality” AND “health”, seven articles were highlighted in the WOS core collection database (accessed on 1 April 2022) for macro and micro analysis.

## 3. Results

### 3.1. Social Mentality and Health in the China National Knowledge Infrastructure (CNKI)

#### 3.1.1. Macro Analysis Results

In this paper, the CNKI academic journal database has been used as the data source and the themes were “social mentality” and “health”. The time span of the literature retrieval was set to all years. The SCI, EI, Peking University Core Journals, CSSCI, and CSCD were selected as the sources. A total of 120 articles have been obtained, of which 117 related articles were selected for bibliometric analysis.

**Figure 2:**
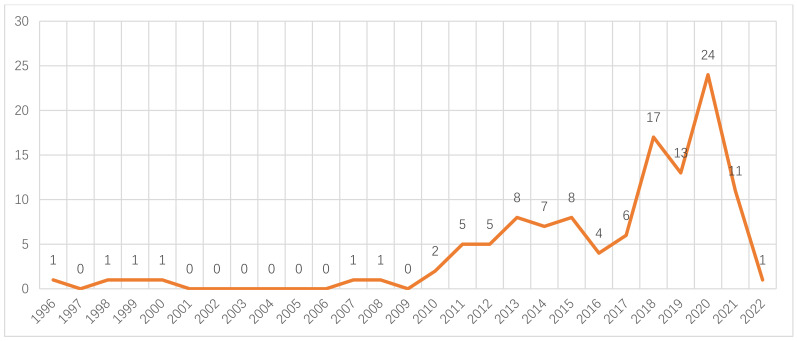
The number of articles on the theme of “social mentality” and “health” in the China National Knowledge Infrastructure (CNKI) database before 1 April 2022 (generated by authors).

As shown in Figure 2, the research on social mentality and health started in the year 1996. Before the year 2010, the annual number of published articles was less than two. Since the year 2010, the annual number of published articles has increased significantly and remained above four, and it reached the peak (24 articles) in the year 2020. Interestingly, in the following year, 2021, the number of articles was reduced to 11, less than the half of those in 2020. However, the research on social mentality and health in the CNKI database is currently in a state of flux, but it is still hot.

1. Keyword co-occurrence analysis

**Figure 3:**
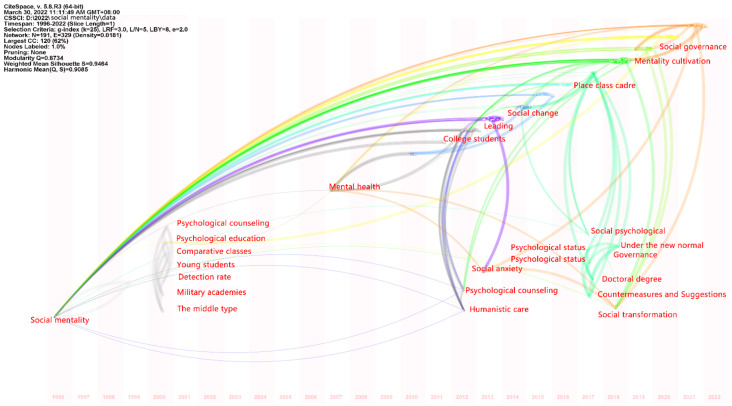
Time zone diagram of keywords on social mentality and health in the CNKI database before 1 April 2022 by keyword co-occurrence analysis via CiteSpace software (generated by the authors). The interpreted English words and phrases are in line with the original Chinese words and phrases in CiteSpace software.

In the setting part, Years Per Slice was set to ‘1’and Node Type was set to ‘Keywords’, resulting in 191 nodes and 329 connections, and we selected ‘Timezone view’ in Layout to get the time zone diagram, as shown in Figure 3.

As shown in Table 1, ‘social mentality’ is still the keyword with the highest frequency of 55 times and the highest centrality at 0.72, while the centrality of ‘humanistic care’, ‘social psychology’ and ‘youth’ are 0. Among the TOP 12 keywords, nine have been studied in the past 10 years. As shown in Figure 3, the research on social mentality and health first appeared in 1996. Then in 2000, the keywords ‘psychological counseling’, ‘psychological education’, and ‘mental health’ became the research direction with the establishment of a mental health research framework based on ‘psychological counseling’ and ‘psychological education’. In 2012, ‘college students’ became a hot research object and more attention was paid to ‘humanistic care’ in social mentality. In the recent 10 years from 2013 to 2022, the psychological state of mental health represented by ‘social anxiety’ has become a major research focus. In addition, social mentality and health have a strong relationship with keywords that are closely related to social change, social transformation, social governance, and political policy, as shown in Figure 3.

2. Keyword clustering analysis

**Figure 4:**
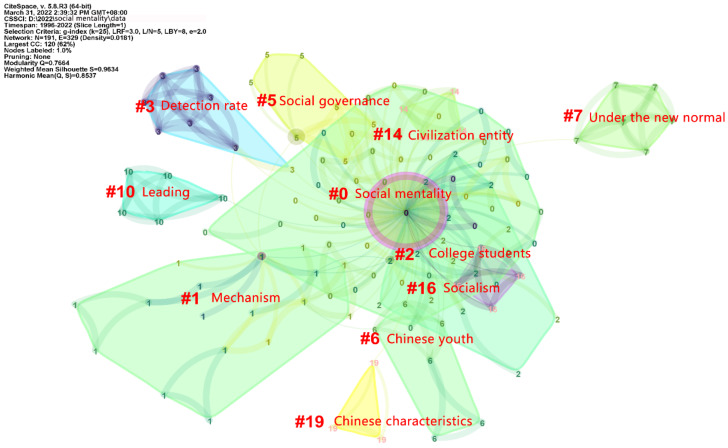
Clustering map of keywords on social mentality and health in the CNKI database before 1 April 2022 by keyword co-occurrence analysis via CiteSpace software (generated by the authors). The interpreted English words and phrases are in line with the original Chinese words and phrases in CiteSpace software.

The CiteSpace software provides two indicators, i.e., the modularity Q value (Q value) and the mean silhouette value (S value), in line with the network structure and clustering clarity, which can be used as a basis to evaluate the mapping effect [45]. After the cluster analysis via CiteSpace software on the 117 articles, 11 clusters including cluster 0 ‘Social mentality’, cluster 1 ‘Mechanism’, cluster 2 ‘College students’, cluster 3 ‘Detection rate’, cluster 5 ‘Social mentality’, cluster 6 ‘Chinese youth’, cluster 7 ‘Under the new normal’, cluster 10 ‘Leading’, cluster 14 ‘Civilization entity’, cluster 16 ‘Socialism’, and cluster 19 ‘Chinese characteristics’ have been generated, as shown in Figure 4, in which modularity Q = 0.7664 > 0.3 and mean silhouette S = 0.9634 > 0.7, indicating that the structure of the cluster is significant and the clustering is convincing [45]. In the 27 years of studies from 1996 to 2022, nearly all the clusters except cluster 7, ‘Under the new normal’, are more closely related to each other, as shown in Figure 4. The new normal is accompanied by new problems, new contradictions, and potential risks, which inevitably affects people’s social mentality [46], and is an environmental state. Results in Table 2 reveal that, excluding cluster 3 ‘Detection rate’ and cluster 16 ‘Socialism’, the average years of the most clusters are all studied after 2013. Results in Figure 4 and Table 2 indicate that among studies centered on cluster 0 ‘Social mentality’ and cluster 1 ‘Mechanism’ are associated with the causes of health problems related to social mentality [47], while cluster 3 ‘Detection rate’ facilitates people’s health from the perspective of psychological education and treatment. In the new era of cluster 7 ‘Under the new normal’ and cluster 19 ‘Chinese characteristics’, ethnic minority areas are regarded as a new research direction, while cluster 2 ‘College students’ is still the most important factor in the social mentality and health research group. At the same time, cluster 6 ‘Chinese youth’ also further emphasizes the importance of research on the social mentality of young people. Cluster 10 ‘Leading’ indicates that the leading of a core value in the field of values is an inevitable requirement to cultivate a healthy social mentality [48]. In addition, cluster 5 ‘Social governance’ and cluster 16 ‘Socialism’, related to political policy, are also important factors affecting social mentality and health. Furthermore, cluster 14 ‘Civilization entity’ refers to a community of knowledge, beliefs, norms, and ideas, which emphasizes people’s living state, emotional mode, values, and behavior mode [49], and among which social mentality will often rise in a long period of time and precipitate into concrete civilization consciousness and the subjectivity consciousness of a civilization entity [50].

#### 3.1.2. Macro Analysis Results

After macro quantitative analysis, micro qualitative analysis was conducted according to the results of macro quantitative analysis, from which further classification has been carried out to reveal the development status and future development trend of social mentality and health. In the macro quantitative analysis, as shown in Figure 4, cluster 0 ‘Social mentality’, cluster 1 ‘Mechanism’, cluster 3 ‘Detection rate’, cluster 5 ‘Social mentality’, cluster 10 ‘Leading’, cluster 14 ‘Civilization entity’, and cluster 16 ‘Socialism’ are all about social mentality and social mental health, while cluster 2 ‘College students’, cluster 6 ‘Chinese youth’, cluster 7 ‘Under the new normal’, and cluster 19 ‘Chinese characteristics’ are further analyses for different groups. Therefore, a follow-up micro qualitative analysis has been employed to further investigate in-depth phenomena on social mentality and health.

1. Social mentality and social mental health

A healthy body, a well state of mind, and a sound personality are the important signs of physical and mental health for people and are the internal premise to form a harmonious society [51]. Hence, it is necessary to make efforts to build a healthy social mentality, which is the fundamental requirement of social harmony [52]. As indicated in Table 3, nine studies clearly indicate the implemented research methods, including empirical analysis [53], literature review [54], questionnaire [55,56,57], case study [58,59], text analysis [60], and comparative research and comparative statistics [61].

The research on social mentality and social mental health mainly focuses on the current research status of social mentality, current social psychology, and social mentality, as well as the cultivation of a healthy social mentality. The cultivation of a healthy social mentality needs to be carried out from five aspects: culture, sports, public opinion, mental health, and a system of public psychological services (SPPS). There are nine studies that have explored the background of the development of social mentality from various perspectives such as social transformation, while 17 studies have showed the current status of social mentality, especially in the context of the epidemic, and the remaining 49 studies have revealed the cultivation methods of a healthy social mentality from different perspectives. As shown in Table 3, Yu has conducted four studies on mental health [62,63], COVID-19 [64], and social psychological services [65]. Wang also has four studies in the field of social transformation background [56], sudden wealth [66], social mentality uncertainty [67], and SPPS [68]. In addition, Bian has used questionnaires and empirical analysis to analyze the background of the development of social mentality under the epidemic situation [57] and employed sports to cultivate a healthy social mentality [53]. Furthermore, Lu [69,70] has written two articles on the SPPS in 2020, and Si’s two studies [71,72] also focused on the cultivation of a healthy social mentality.

**Table 3:** Statistics of clusters on social psychology and healthy social mentality in the CNKI before 1 April 2022 (generated by the authors).

Content	Count	Year	Author	Method
Social mentality cultivation/System of public psychological services (SPPS)	47	2021	Zhu et al. [73]	-
Social mentality cultivation/SPPS		2021	Miao [74]	-
Social mentality cultivation/Sports		2021	Bian et al. [53]	Empirical analysis
Social mentality cultivation/SPPS		2020	Wang [68]	-
Social mentality cultivation/SPPS		2020	Lu et al. [69]	-
Social mentality cultivation/SPPS		2020	Ge et al. [75]	-
Social mentality cultivation/SPPS		2020	Zhang et al. [76]	-
Social mentality cultivation/Sports/Fitness		2020	Wang [77]	-
Social mentality cultivation/SPPS/COVID-19		2020	Xu et al. [78]	-
Social mentality cultivation/SPPS		2020	Xiao [79]	-
Social mentality cultivation/SPPS		2020	Lu et al. [70]	-
Social mentality cultivation/SPPS		2020	Huang et al. [80]	-
Social mentality cultivation/SPPS		2020	Hou [81]	-
Social mentality cultivation/COVID-19		2020	Liu et al. [82]	-
Social mentality cultivation/Sports		2019	Han et al. [54]	Literature review
Social mentality cultivation/SPPS		2019	Lin et al. [58]	Case study
Social mentality cultivation/SPPS		2019	Chi et al. [60]	Text analysis/Case study
Social mentality cultivation/Values		2019	Wang [83]	-
Social mentality cultivation/Online public opinion		2019	Sun [84]	-
Social mentality cultivation/SPPS		2018	Fu [85]	-
Social mentality cultivation/Psychosocial environment		2018	Si et al. [71]	-
Social mentality cultivation		2018	Si [72]	-
Social mentality cultivation		2018	Li [86]	-
Social mentality cultivation/SPPS		2018	Lv et al. [87]	-
Social mentality cultivation		2018	Chen [88]	-
Social mentality cultivation/Values		2017	Chen [89]	-
Social mentality background/Social transformation		2017	Yu [65]	-
Social mentality cultivation/Network governance		2017	Yu et al. [90]	-
Social mentality cultivation/Values		2016	Sun [91]	-
Social mentality cultivation/Online public opinion		2016	Du [92]	-
Social mentality cultivation		2015	Feng [93]	-
Social environment/Social mentality cultivation		2015	Chen [94]	-
Social mentality cultivation/Values		2015	Mei et al. [95]	-
Social mentality cultivation/Values		2015	Wang et al. [96]	-
Chinese spiritual/Social mentality cultivation		2014	Chen [97]	-
Social mentality background/Social reform		2014	Tang et al. [98]	-
Social mentality cultivation		2013	Zeng [99]	-
Social mentality cultivation		2013	Chen et al. [100]	-
Social mentality cultivation/Culture		2013	Liu [101]	-
Social mentality cultivation		2012	Zhang [102]	-
Social mentality and social management		2012	[103]	-
Social mentality cultivation		2012	Qiu et al. [104]	-
Social mentality cultivation/Media public opinion		2012	Xu [105]	-
Social mentality cultivation/Sports		2011	Zou et al. [106]	-
Social mentality cultivation/Culture		2011	Ding [61]	Comparative research and comparative statistics
Social mentality cultivation/Culture		2011	Xu [107]	-
Social mentality cultivation/Public opinion		1999	Teng [108]	-
Social psychology/Social mentality	17	2022	Chen [109]	
Social psychology/Social mentality		2021	Yu [62]	-
Social psychology/Social mentality		2020	Wang et al. [110]	-
COVID-19		2020	Wu et al. [59]	Case study
COVID-19/Emotion		2020	Guo et al. [55]	Questionnaire
COVID-19		2020	Zhao [111]	-
COVID-19		2020	Yu et al. [64]	-
COVID-19		2020	Bian [57]	Questionnaire
Social mentality guidance/COVID-19		2020	Chen [112]	-
Mental health		2019	Yu et al. [63]	-
Social psychology/Social mentality		2019	Wu [113]	-
Social psychology/Social mentality		2018	Wang [66]	-
Social psychology/Social mentality		2017	Xue et al. [114]	-
Social mentality/Uncertainty analysis		2016	Wang [67]	-
Psychological Counseling/Social mentality		2014	Xie et al. [115]	-
Social psychology/Social mentality		2014	Zhu [116]	-
Social psychology/Social mentality		2013	Liu [117]	-
Social mentality background/Social transformation	9	2020	Jiang et al. [118]	-
Social mentality background/Social transformation		2020	Wang et al. [56]	Questionnaire
Social mentality background/Social transformation		2015	Lv [119]	-
Social mentality background/Social risk management		2015	Li et al. [120]	-
Social mentality background/Social psychology		2014	Zeng et al. [121]	-
Social mentality background/Social transformation		2011	Jiang et al. [122]	-
Physical and mental health/Harmonious society construction		2010	Chen et al. [51]	-
Harmonious society construction		2007	Wu [52]	-
Socialism and market economy		1996	- [123]	-

- Current research status of social mentality

Wang et al. [56] employed a questionnaire survey to study the need for a better life as an indicator of social mentality and developed a measuring tool for a better life according to the structure of the need for a better life, which compares people’s yearnings for a better life with their evaluation of their current needs for a better life and finds that the sense of fairness, mental health, life pressure, and social governance environment are the main factors affecting the satisfaction of people’s needs for a better life. During the period of social transformation in China, with the deepening of reform and opening up and the establishment of a market economic system, China’s traditional interest pattern is undergoing a revolutionary adjustment [122], which requires both fairness and efficiency [123]. With the emergence of problems such as the gap between the rich and the poor, social antagonism, and judicial injustice, the social mentality will become more unstable [121]. Thus, China needs a healthy social mentality to match it, so that the society can run well [123]. At the same time of social transformation, social mentality is also transformed and reorganized [122]. In the process of comprehensively deepening the reform of further promoting economic development, there are some outstanding characteristics of social mentality that directly affect the development of people’s livelihoods [98]. Moreover, in this period of strategic opportunity, social risks are in a high incidence stage, and social risk governance not only needs to rely on the ‘hard governance’ of the government, but also needs ‘soft governance’ from the perspective of psychological security [120]. At present, the main contradiction in Chinese society has shifted to the contradiction between the growing needs for a better life and the unbalanced and inadequate development, which indicates that cultivating a healthy social mentality and improving people’s sense of gain and happiness have become the needs of the people, society, and the country [118]. Therefore, the content of people’s increasing needs for a better life is an important topic in the study of social mentality [56].

- Current social psychology and social mentality

Social mentality can not only reflect various social problems in the process of social change, but also help to understand the social situation, public opinion, social hotspots, and social emotions, so as to achieve a positive and healthy social mentality and promote the operation of society [119]. Additionally, social mentality comes from individual psychology but exists in the form of the whole society. It is a psychological state that can reflect the trend of people’s thoughts or tendencies in a certain stage of social development, and it is the refraction of social reality [115]. Hence, the study of social psychology is also part of a healthy social mentality. The current studies mainly focus on the negative psychological states of impetuosity [114,116], anxiety [62,113,117], and desire for quick success and instant benefit [66]. Social impetuosity is also a kind of negative mental state widely existing in the social changes [116]. Xue et al. [114] believe that the unhealthy mental state of being popular overnight by playing ugly is the root of anxiety, impetuosity, distress, and other unhealthy social mentalities. Moreover, education [113], income, job competition, the pursuit of fame and wealth, and some groups of mental health problems are the causes of anxiety [62,117]. Furthermore, social anxiety gradually shifted from the individual to the group [117].

With COVID-19, people’s levels of stress, anxiety, and depression have risen further [124]. In a further study of the COVID-19 epidemic, Guo et al. [55] analyze the emotional status and social mentality in the Chinese public through the data from two online public questionnaire surveys, which reveal that in terms of social mentality, part of the public at different psychological stages tends to be depressed and angry. In addition, Wu et al. [59] study the epidemic situation and emotions in a community in Anhui province, China, and find that the restructuring of psychological space deepens the epidemic fear and social mentality crisis, which poses a severe challenge to China’s urbanization and living environment. The normalization of epidemic prevention and control has become the consensus of the whole society in China, in which a healthy social mentality is an important support to win the battle against the epidemic [64]. Interestingly, Bian’s [57] research on people’s social mentality under the COVID-19 epidemic through questionnaires shows that although the effectiveness of physical isolation has a significant negative effect on social mentality, the better the epidemic prevention behavior is, the more people tend to have a healthy mentality. Furthermore, in terms of mental health, although the mental health of urban residents in China is not optimistic, the mental health of the majority of the population is improving year by year [110]. After COVID-19, the Chinese people and the government will pay more attention to mental health and social psychology [109].

- Cultivation of healthy social mentality

Social mentality can influence people’s behavior and social development to a certain extent [104]. The cultivation of a positive social mentality needs to be carried out from the aspects of culture, sports, public opinion, and mental health, and the construction of SPPS is one important approach to achieve it.

(1) Cultural perspective

From the perspective of cultivating a healthy social mentality, Ding [61] conducts a comparative statistics study on the data in a number of years, which indicates that people’s cultural behaviors could reflect the development of a social cultural mentality. In addition, in the face of the current situation of COVID-19, there is a long way to go to cultivate a positive and healthy social mentality. Strengthening cultural construction and enhancing cultural power is an effective approach to cultivate a positive and healthy social mentality [107]. Under the guidance of advanced culture, the public will be able to view the contradictions and problems in China’s economic and social development in a correct way, inspire the spirit of enterprising and tenacious struggle, and build self-esteem, rationality, positivity, and a healthy social mentality [101].

(2) Sports perspective

Bian et al. adopt a series of cutting-edge empirical analysis methods in the field of computational sociology, such as time-series analysis, emotion analysis, panel data regression, and mediation effect tests, and reveal that online fitness can significantly increase positive attitudes even when online social interaction is controlled [53]. Interestingly, with sport as an active approach to social culture, social mentality is constructed via sport not only through the mutual construction of a collective and individual in social culture, but also by the unique visual representation characteristics of sports itself [77]. According to the study of Zou et al. [106], sport plays a positive role in stabilizing the primary social mentality, while through a literature review, Han et al. [54] summarize that sports can promote people’s happiness, interpersonal trust, national identity, negative emotions, pro-social behavior intention, self-efficacy, and other social psychology indicators.

(3) Public opinion perspective

From the perspective of public opinions, emotions among groups are more likely to infect each other and form a diffusion effect because of sensibility rather than the rationality of social mentality [108], in which the media has the responsibility to play the leading role in the core value system of society toward a healthy social mentality [105]. In addition, the media’s three-dimensional report and health report system construction in line with social mentality will become the main approaches participating in risk governance [82]. However, online secondary public opinion weakens the influence of the mainstream media and may form an extreme social mentality, resulting in excessive emotional catharsis [84]. Therefore, it has become an important theoretical and practical issue in China to innovate social management and cultivate a healthy and progressive network for the public opinion environment [92]. The cultivation of a healthy social mentality by public opinion needs to ensure the right for the public to know and the correct channels of appeal, and strengthen the scientific guidance of media information, in which the government should make full use of the internet to popularize mental health knowledge among social participants [90].

(4) Mental health perspective

From the perspective of mental health, the goal of improving the social mental health education and service system is to cultivate a good social mentality [125]. At present, it is necessary to form a positive and healthy social psychology environment, enhance people’s sense of social psychology security, and strengthen the forward-looking guidance toward a social mentality [71]. Yu et al. [63] put forward the concept of great mental health education, which starts from holistic and developmental ideas by fully considering China’s social environment and the development characteristics of the new era and establishes the concept of a mental health education system in line with China’s national conditions and full of Chinese characteristics to cultivate a positive social mentality.

(5) The perspective of the system of public psychological services (SPPS)

The creation of a healthy social mentality is about establishing the SPPS, of which the formation of a good social mentality is an important goal [73]. By using a case study to investigate the construction of public psychological services in Changning District, Shanghai, Lin et al. stress that (1) the SPPS is not equal to mental health services, and the public psychological services are not only for individuals, but also for the whole society; and (2) on the basis of providing mental health services for individuals, the service system regulates social mentality and social emotions through public policies and social governance, and should be implemented with big data technology for social mentality monitoring [58].

The main contents of the service system include three modules: mental health service, cultivation of social mentality, and construction of community identity, the main functions of which are, respectively, the prevention and treatment of psychological diseases, improvement of the mental health levels across China, and cultivation of self-esteem, rationality, positivity, and a healthy social mentality, as well as shaping the unified cultural identity of the Chinese nation and the identity of a community with a shared future for mankind [87]. The Chinese government further emphasizes that the service system will be further improved, including characteristics such as the cultivation of self-esteem, self-confidence, rationality, composure, and optimism, among people [85]. As such, the studies and practices of these aspects have attracted more and more attention. Additionally, it is an important means and content of social governance in the new era [69].

Through text analysis on network text data of the actual work in the construction of the SPPS, Chi et al. [60] find that there is a large gap between the theory and practice of constructing the service system in China, and the current construction practices mainly concern the level of mental health services and social risk prevention and control. At present, many studies have put forward different views on the construction of the service system. In terms of perspective and population, people should base themselves on the whole population group and carry out service activities in the whole life cycle stage [75,79]. In terms of content, the mechanism of prevention and intervention of public psychological services should be strengthened [81], and the service tasks for the prevention and treatment of psychological diseases to the comprehensive satisfaction of various psychological needs should be expanded [79]. In terms of implementation, various approaches should be carried out in parallel [75], in which the main body of implementation should be switched from professionals to the whole society [79], and the integration of resources and approaches should be emphasized [80]. Based on localization, the public psychological service network should be built while the establishment of community psychological service is being optimized [81]. The arrival of COVID-19 has also further promoted the establishment of social and psychological services, and helped the public and government to make more rational decisions [78]. However, the current research on the SPPS is not sufficient, lacking a consistent understanding of the scientific connotation and basic structure of the service system [70]. Although the two contexts of service system construction are social mentality as the main line and mental health as the secondary line, the current practices on the SPPS are carried out in the secondary line, mainly because there are no theories and cases that can be used for reference [68]. This further reflects the problems of insufficient psychological support and personnel training in establishing a social psychological service [74]. The inadequacy and shortcomings of such implementations have been further highlighted in the context of COVID-19 [76].

Social psychology and social mentality are closely related, and a change of social psychology will affect the social mentality. The cultivation of a social mentality can be carried out from the aspects of culture, sports, public opinion, and mental health. The SPPS is also an important approach to cultivate a healthy social mentality. However, relevant theories are still under construction at present, and there is a gap of relevant talents, which leads to a deficiency in constructing the SPPS. This is a research direction that needs to be further explored and supplemented in the future.

2. Population and healthy social mentality

The absorption, internalization, and reaction of various social groups and classes to the same social reality are not exactly the same [126]. Thus, it is necessary to analyze the social mentality to the social groups and classes. As shown in Table 4, the research methods used to study the social groups and classes with a healthy social mentality are mainly questionnaires [127,128,129] and interviews [130,131], of which five studies clearly state their research methods. The current studies on population and a healthy social mentality are mainly focused on following identified social population groups: youth (including small-town youth, college students, youth-related teachers, young medical workers, and new migrant workers) and ethnic minority areas and border areas, as well as soldiers, cadres, and laid-off workers. There are 12 direct studies on youth including small-town youth and 17 studies on college students, one study on young medical workers, and two studies on the new generation of migrant workers. In addition, there are four studies on teachers closely related to college students. Furthermore, people from ethnic minority areas and border areas have received attention, with five studies, while cadres, soldiers, and laid-off worker groups have fewer studies, with one study for the each group. However, no author has more than two articles in the field of social mentality and population research.

- The social mentality of youth

As the backbone of social development and the reserved force of national construction, the healthy development of a social mentality for youth is particularly crucial. It is a great practical significance for the development of the whole society and national governance to understand the current social mentality of young people and to explore the effective approaches to cultivate healthy youth in terms of social mentality [155], which also has an important impact on the growth of young people [154]. For further study of youth’s social mentality, current youth with some typical mentality are more and more obvious, such as Buddha-like youth [153], empty-nest youth [148], and ‘emo’ culture that is a kind of ‘brocade carp’ culture and embarrassing culture [147]. Young people have being in these passive states to be subjectively unwilling to struggle for a long time, and be objectively manifested as self-mockery and self-deprecation, and meanwhile actually resisting external evaluation, which is very unfavorable to the healthy development of young people’s physical and mental health [150]. The main factors affecting the healthy development of youth’s social mentality are the impact of a complex social trend of thought on youth’s social mentality, the disturbance of some new media in the information age, and the lack of youth’s quality psychological education [152].

(1) The social mentality of small-town youth and college students

Youth is a huge group, and current studies have carried out in-depth analyses on the various youth groups. Wang et al.’s [131] study on small-town youth through an online questionnaire surveys and face-to-face interviews finds that their class identity is low; their evaluation of social fairness, morality, and trust are generally not high; they are mostly concerned about the gap between the rich and the poor, medical care, and education; and they generally have high work pressure, prominent mental health problems, and low subjective well-being. Another large research group is young college students, whose mental health problems and illnesses are getting more attention [134]. At present, there are several kinds of college students with ideological confusion, psychological distress, employment difficulties, and learning difficulties [143]. College students’ psychological problems vary from person to person, in which a healthy individual psychology is an important part of positive social mentality [134]. The psychological changes of college students brought by an increasing sense of injustice and the lack of social integrity are not optimistic [143]. In general, anxiety and impetuosity are prominent in college students’ social mentality. Hence, it is very important to cultivate the healthy mentality of college students. There are various approaches to cultivate a healthy social mentality for college students. First, as described in Section 3.1.1, cultivating and practicing core values is still an important approach for young college students to have a healthy social mentality [131]. Second, young college students need to have a clear position on their own, strengthen their self-cultivation, cultivate their own dialectical thinking [143], and pay attention to self-regulation [144]. Third, colleges should strengthen the education of students’ ideals and beliefs [143], implement theory with practice [135], strengthen poverty funding [156], establish a platform for the college students to start their own businesses [142], coordinate various forces to relieve college students’ employment pressures [156], promote the construction of the college’s psychological curriculum education system [136], and improve the implementation system of psychological education [133]. In addition, colleges and universities should strengthen the research on the problems of college students, provide theoretical support for the cultivation of healthy social mentality of college students, improve the SPPS, and form a comprehensive three-dimensional cultivation path [156]. For instance, mental health education is permeated into music education [164]. In the context of COVID-19, it is even more important for colleges and universities to guide the positive social mentality of college students [124]. Fourth, in terms of public opinion toward the students, the government should adhere to a people-centered policy, society should achieve equity and justice, and the media should play a guiding role [135], which are used to ease social emotions [138], master the discourse power of network ideology [137], strengthen the construction of new media communication platform for mainstream ideology, enhance the cohesion and influence of campus network opinion leaders [140], and create a positive and harmonious social public opinion environment [136], forming an all-round three-dimensional cultivation path, through improving the SPPS [132]. Within the family and friends of college students, equal communication should be advocated in the family [135] and the growth space of college students’ social mentality should be shaped by the power of peer education [137]. In general, the cultivation of a healthy social mentality in college students needs to pay attention to the system’s construction and provide integrated roles for the college, family, and society [144]. Interestingly, vocational college students and postgraduates have been focused on, and it is believed that school-enterprise cooperation can increase students’ practice, and psychological problems can be found and solved in the process [141], in which the postgraduates should be paid attention to via the strengthening of the construction of an early-warning system for social mentality in building a positive social mentality of postgraduates [112].

(2) The social mentality of teachers

College teachers, who are not only disseminators of knowledge but also shapers and leaders of the students’ values, are closely related to the construction of a healthy social mentality in young college students [162]. The healthy social mentality of college teachers is not only the objective requirement to fulfill the responsibility of moral education, but is also the realistic need to satisfy personal happiness [163]. Therefore, it is necessary to strengthen the teachers’ consciousness of responsibility, adjust their social mentality, create a fairer teaching environment for them, and provide them with a positive and healthy attitude, so as to provide proper guidance for students’ future development [161].

(3) The social mentality of young medical workers and the new generation of migrant workers

In the current studies on other youth groups such as young medical workers and the new generation of migrant workers, Bi [130] studies young medical workers in a hospital in Tianjin, China, by using a questionnaire survey and interviews, and suggests that with the deepening of social changes and medical system reform, the social mentality of young medical workers shows a new feature of accelerating change becoming increasingly complex. Thus, it is necessary to broaden the channels for the expression of young medical workers’ demands and strengthen the guidance of their mentality for them to build a healthy social mentality. In addition, the social mentality of the new generation of migrant workers has gradually attracted attention. The healthy social mentality of the new generation of migrant workers is an important condition for promoting the construction of a political consciousness civilization [166]. At present, the new generation of migrant workers is under great pressure to survive in a state of overall anxiety [166], lacking a sense of belonging to the city, with a weak sense of fairness and a strong need for dignity, and the high expectation of personal development contrasts with reality [165]. Hence, it is necessary to create a fair and institutional environment, construct a new and effective social psychological counseling mechanism, establish and improve the voice mechanism of migrant workers’ demands, and strengthen the construction of a social support system for the psychological problems of the new generation of migrant workers [165].

- The social mentality in ethnic minority areas and border areas

The construction of a healthy Chinese national mentality of border nationalities is of great significance to the construction of a community consciousness of the Chinese nation [160]. La [129] conducts an online questionnaire survey to obtain a preliminary understanding of the social mentality and psychological stress of people in Qinghai province, China, with the background of COVID-19, which shows that there are general changes in people’s physical and mental health, and, as such, attention should be paid to observing and helping the mentality of vulnerable people after the epidemic. Guan et al. [159] believe that the importance of ethnic psychological problems and the urgency of solving them means the current work must pay attention to the construction of SPPS in ethnic minority areas. However, at present, there is insufficient understanding of public psychological services in minority areas, a shortage of public psychological service personnel in minority areas, a relatively backward public psychological service infrastructure in minority areas [157], a weak willingness to accept services, and the chimeric problems of ethnic religious beliefs and social psychological construction [159]. Therefore, the SPPS in ethnic areas in the new era provides a new scheme with Chinese characteristics for solving ethnic problems and innovating the cause of national unity and progress based on the national conditions of China, with the traditional cultures and psychological behavior characteristics of each ethnic group and the comprehensive use of multidisciplinary theories and strategies [158].

- The social mentality of soldiers, cadres, and laid-off workers

Feng et al.’s [127] research on soldiers’ mentality by means of a questionnaire survey indicates that improving mental health in soldiers is an in-depth need of military construction in the new century and a new content and topic for personnel training in military academies. In addition, Yan’s [128] questionnaire on cadres’ social mentality suggests that the construction of social mentality must focus on leading cadres, the ‘key minority’, and good cadre mentality will set a good vane for guiding the overall social mentality. Furthermore, for laid-off workers, it is necessary to comprehensively adjust and balance through a variety of ways to eliminate psychological barriers, establish a positive and rational social mentality, and then promote social stability [167].

The current crowd is mainly focused on the youth group, but the construction of a harmonious society needs the guarantee of a healthy social mentality of all people; as such, it is necessary to explore more social groups and classes, especially vulnerable groups and groups with weak discourse voices.

### 3.2. Social Mentality and Health in Global Web of Science Core Database (WOS)

A literature search was conducted for all years with the themes (“Social mentality” and health*) in the core collection of the WOS database, resulting in the seven relevant articles that have been listed in Table 5. Since there are an insufficient number of articles, the co-occurrence network analysis cannot be formed via CiteSpace software.

In terms of research methods, the studies in the WOS core collection database mainly employ questionnaires [4,34,169,171] to investigate social mentality to aid health, followed by scales [168,172] and participatory action research methods [170]. Santos et al. [168] use scales on the basis of comparative study, while Kou et al.’s [170] study on gardening and mental health adopts a participatory action research method. As shown in Table 5, the studies on health and social mentality are the current research hotspots, which first appeared in the year 2015, and the remaining six studies were all published in the three recent years (2020 to 2022). In terms of the research keywords, ‘COVID-19’ and ‘pandemic’ have been embedded in most of the studies, followed by ‘mental health’. Based on the factors influencing people’s social mentality during the epidemic, Nie et al.’s study [171] suggests that the epidemic-related factors, e.g., self-efficacy, gender, education level, age, risk level, and knowledge, and objective social support are significant predictors of public panic. Wang et al. [4] further explore the important effects of social support, epidemic knowledge, self-efficacy, and risk on mental health during COVID-19. Interestingly, Zhao et al. [34] find that college students’ social mentality decreased during the peak period of COVID-19 and increased during the controllable risk period. For the construction of a healthy social mentality, Xi et al. [169] believe that social support and external assistance, as well as positive interpersonal interaction experiences, are crucial to the cultivation of personal peace of mind and the improvement of happiness in the workplace. In addition, Kou et al. [170] believe that community gardening is an important approach to improve people’s mental health in the context of COVID-19. The above studies indicate that social mentality and health, especially mental health, in the context of COVID-19 are important and worth-exploring areas, in which some studies looked at occupational mental health [172], particularly within the caregiver profession [168].

## 4. Discussion

### 4.1. Hot Topic in Social Mentality and Health

The results of Section 3.1.1 and Section 3.2 indicate that social mentality and health is growing in China and around the world. In addition, the results of high-frequency keywords in Section 3.1.1 echo that the social environment is an important factor affecting social mentality [173]. The current study on social mentality and social mental health mainly focuses on the current research status of social mentality, current social psychology, and social mentality, as well as on the cultivation of a healthy social mentality. In the context of a rapidly changing social environment, especially in the context of COVID-19, people lack a sense of security and have an unhealthy social mentality [174]. An unhealthy social mentality has considerable influence on social interpersonal relationships, daily life, the economy, culture, and governance [175]. Importantly, the cultivation of a healthy social mentality is associated with culture, sports, public opinion, mental health, and the SPPS, in which the subjective initiative in the population of young people is a key group for a healthy social mentality [176]. Furthermore, results in Section 3.2 suggest that in the global WOS database the current studies on social mentality in the context of COVID-19 take a dominant position, and the main methods such as social support, external assistance, positive interpersonal interaction experience, and gardening are proposed to actively explore the cultivation of a healthy social mentality. Thus, social-mentality-assisted health could facilitate with gradually achieving SDG 3, which aims to ensure healthy lives and well-being for all.

Furthermore, the results of Section 3.1.2 and Section 3.2 regarding research methods used for studying social mentality and health suggest that the studies in the CNKI database tend to employ a speculative method (qualitative research) and an empirical method (quantitative research) [177]. The questionnaire represented by the social mentality scale is an important method to study social mentality at present, which is proposed by referring to the theories of others [178,179], while the reliability and validity test instructions of measuring tools are usually not available [180]. In contrast, most of the studies from the WOS database adopt quantitative methods such as questionnaires, scales [181], and comparative studies. Interestingly, Li [182] believes that efficient investigation methods and mathematical analysis skills should be mastered to produce appropriate and standardized investigation reports in the study of social emotions. In addition, Wang et al. [183] use the random forest method to study mental health, and further introduced an artificial intelligence method into the study of social mentality. Furthermore, Lin [58] applies big data technology to monitor the social mentality of the public. Therefore, the research methods for a future social mentality study could be in line with the development of new technologies and multidisciplinary integration, such as artificial intelligence.

### 4.2. Development Trend of Social Mentality and Health

#### 4.2.1. Exploration of New Approaches to Cultivate a Healthy Social Mentality

Among the five aspects to cultivate a healthy social mentality in the results of Section 3.1.2, sport is a relatively new approach. Sport plays a positive and effective role in releasing negative social emotions, suturing social trauma, and shortening unfair social psychology [106]. As a special cultural phenomenon, sport has formed a group and a macroscopic psychological representation in social development, with the psychological discipline provided by ‘collective representation’, in which the psychological states of different sports participants can be coordinated in a short time [77]. Art is often mentioned together with sports, but only one study [164] on music education contributes to the cultivation of a healthy social mentality, which may be due to the variety of people’s expression and understanding of art [184], and to their own independent, private, inexpressible, and intuitive senses [185]. Interestingly, some works of art are created in social relationships and activities, which is a process of constantly creating and maintaining relationships with others [186]. As such, the social nature of this kind of art can be further explored in the context of the definition of social mentality, such as the idea of community gardening as a public health strategy [170]. In addition, art therapy has the function of curing some patients’ physical and psychological diseases [187]. Furthermore, in the construction process of the SPPS, socialized mental health service institutions are likely to become the most dynamic factors in the SPPS [188]. Hence, the promotion of art therapy in the SPPS, as one of the approaches to cultivate a healthy social mentality, could become a potential research direction in the future.

The construction of the SPPS still faces the practical dilemma of outdated ideological cognition, insufficient talent reserve, poor service mode, unclear division of power and responsibility, and lack of financial support [189]. For individuals, the higher the level of social support, the more they are inclined to adopt positive coping styles [190]. In addition, the current practices in establishing the SPPS focus on solving individual mental illness and promoting individual mental health, which is the initial stage to construct the SPPS, rather than a systematic construction [191]. Thus, how to then promote systematically constructing the SPPS for benefiting all people and to facilitate achieving SDG 11, which aims at building sustainable cities and communities, could be an important research goal in the future.

#### 4.2.2. Exploration of New Research Direction to Population and Healthy Social Mentality

Youth is a very important population group for the current studies on social mentality to assist health, especially in the current aging background for youth development city construction. Since young people are the most active manpower in the whole social force, the hope of the country, and the important force of social development, it is crucial to understand and cultivate young people’s healthy social mentality [173]. However, the most current studies focus on the whole group of college students without classifying them, which makes it difficult to take targeted measures to solve the problem of college students’ social mentality [176]. In addition, studies have shown that the current mental health situation of middle school students and young teachers is insufficient, and the mental health of the elderly and migrant workers as the main floating populations need more attention [110]. Furthermore, there is also a lack of comparative studies between different groups [192] or under further subdivision of the same group [176]. In the comparison of global and Chinese population studies related to young people, it has been found that the studies on the social mentality of teachers and caregivers have the same research context. For teachers, it is necessary to establish an effective psychological counseling mechanism to shape the healthy individual mentality of college teachers [163] and further provide scientific guidance for the future development of their students [161]. For caregivers, compassion plays an important role in nursing, and Santos et al. [168] have demonstrated their preliminary evidence for the effectiveness of the compassionate mind training program for care homes, suggesting that the training allows caregivers working in residential youth-care settings to develop a subordinate mentality. Therefore, the comparative study and cross-integration of social mentality between different countries with similar populations and contexts will also be the direction of future research.

Results in Section 3.1 and Section 3.2 suggest that because of the uncertainty of the current COVID-19 and the continuous changes in people’s mental health associated with social mentality, future research could better focus on exploring the influencing factors toward mental health under COVID-19 [172], the research methods on mental health [4,169,171], and the approaches to cultivate a healthy social mentality. To be more precise, future research on social mentality to assist health could tend to explore diversified methods to cultivate a healthy social mentality from the perspective of art, improving the SPPS, strengthening the refinement of population research and the comparative study of populations, and exploring the influencing factors and cultivation methods of a healthy social mentality from the perspective of methodology. Furthermore, it is an important topic to explore new training methods for healthy social mentality in the future, which assist the achievement of SDG 3, ensuring healthy lives and promoting well-being for all at all ages, and SDG 11, building sustainable cities and communities [7].

## 5. Conclusions

This paper is the first attempt to employ a bibliometric method to explore the research hotspots and trends of social mentality and health in the world and China from two databases, namely the Chinese-language CNKI and the English-language WOS, with the following contributions: (1) from the macro and micro perspectives, the social mentality and health in the world and China is explored and identified, in which studies are increasing in terms of using the Chinese language and the English language. Qualitative analysis is mostly used in the studies in the CNKI database, and few quantitative analyses have been used, such as questionnaires. Whilst the studies in the WOS database have all implemented quantitative methods including questionnaires, scales, and comparative study, (2) the CiteSpace bibliometric tool software has been used for keyword co-occurrence and clustering analysis for the macro quantitative investigation of social mentality and health, from which 11 identified current research hotspots, namely social mentality, mechanism, college students, detection rate, social mentality, Chinese youth, under the new normal, leading, civilization entity, socialism, and Chinese characteristics, and future research trends of social mentality and health, are revealed. Subsequently, the studies in the CNKI database and WOS database, respectively, have been compared for micro qualitative analysis; and (3) in terms of research results, the current studies in the CNKI database mainly focus on the macro social environmental factors affecting social mentality, aspects to cultivate a healthy social mentality, namely culture, sports, public opinion, mental health, and SPPS, and population research, such as on young people (including small-town youth, college students, youth-related teachers, young medical workers, and new migrant workers), and ethnic minority areas and border areas, as well as soldiers, cadres, and laid-off workers, while the studies in the WOS database pay more attention to social mentality and health in the context of the COVID-19 epidemic situation and a variety of professions.

In the future, it is necessary to use multidisciplinary research methods based on artificial intelligence and big data technology to comprehensively study social mentality and health from the macro and micro perspectives, and to make more accurate countermeasures, especially in the context of COVID-19, based on the need of SDG 3. In addition, the research on social mentality and health will continue to develop, and the application of art and the improvement of the SPPS will be the direction for investigation, based on which the diversified approaches to cultivate healthy social mentality could be explored in the post-pandemic era. In the research on a healthy social mentality and population, it is necessary to refine the study on the same group and the comparative study between different groups. Hence, future research could explore the influencing factors and cultivation methods for a healthy social mentality from the perspective of methodology and of achieving SDG 3, providing healthy lives and promoting well-being for all at all ages, and SDG 11, building sustainable cities and communities in the post-COVID-19 era, as well as the comparison and integration of social mentality between different countries with similar populations and contexts.

However, the bibliometric method using quantitative analysis based on CiteSpace software in this paper relies on the CNKI and WOS databases. As such, the follow-up research could conduct quantitative analysis of multiple and multi-language databases, including Scopus and ScienceDirect, and explore a practice approach for social mentality to assist health in the context of COVID-19.

## Figures and Tables

**Table 1:** Statistics of keyword frequency in TOP 12 keywords on social mentality and health in the CNKI database before 1 April 2022 by keyword co-occurrence analysis via CiteSpace software (generated by the authors).

Frequency	Centrality	Mean Year	Keyword
55	0.72	1996	Social mentality
11	0.11	2007	Mental health
10	0.01	2012	College students
7	0.04	2019	Social governance
5	0.01	2012	Psychological counseling
4	0.02	2017	Social transformation
4	0.01	2012	Cultivation
4	0	2012	Humanistic care
3	0.08	2000	Psychological education
3	0	2017	Social psychological
3	0.03	2021	Ethnic minority areas
3	0	2018	Youth

**Table 2:** Statistics of clusters on social mentality and health in the CNKI database before 1 April 2022 by keyword co-occurrence analysis via CiteSpace software (generated by the authors).

Cluster ID	Size	Silhouette	Mean Year	Cluster Name	Highest Frequency Keyword
0	45	0.965	2015	Social mentality	Social mentality
1	17	0.949	2014	Mechanism	Mental health
2	15	0.898	2014	College students	College students
3	8	0.986	2002	Detection rate	Psychological education
5	7	0.985	2020	Social governance	Social governance
6	6	0.98	2015	Chinese youth	Social psychology
7	6	0.998	2017	Under the new normal	Countermeasures and suggestions
10	5	0.997	2013	Leading	Leading
14	4	0.992	2018	Civilization entity	Deep motivation
16	4	1	1996	Socialism	Market economy
19	3	0.989	2021	Chinese characteristics	Ethnic minority areas

**Table 4:** Statistics on clusters of population and healthy social mentality in the CNKI before 1 April 2022 (generated by the authors).

Content	Count	Year	Author	Method
College students	17	2021	Dong [132]	-
College students		2021	Xu et al. [133]	-
College students		2021	Tan [134]	-
COVID-19/College students		2020	Chen [124]	-
College students		2019	Wang et al. [135]	-
College students		2019	Zhang [136]	-
College students		2018	Wang et al. [137]	-
College students		2018	Liu et al. [138]	-
College students		2018	Wang et al. [139]	-
College students		2018	Li [140]	-
College students		2015	Wang [141]	-
College students		2015	Fu [142]	-
College students		2014	Jin [143]	-
College students		2014	Yu et al. [126]	-
College students		2013	Hu et al. [144]	-
College students		2012	Peng [145]	-
Students		2010	Xiao [146]	-
Youth	12	2021	Zhang [147]	-
Youth		2020	Zhou [148]	-
Small-town youth		2019	Zhao et al. [131]	Questionnaire and interview
Youth		2019	Yuan et al. [149]	-
Youth		2019	Wang [150]	-
Youth		2018	Chen [50]	-
Youth		2018	Du et al. [151]	-
Youth		2018	Li [152]	-
Youth		2018	Pu et al. [153]	-
Youth		2018	Jiang [154]	-
Youth		2017	Hu [155]	-
Youth		2014	Zhu [156]	-
Ethnic minority areas	5	2021	Li et al. [157]	-
Ethnic minority areas		2021	Wang et al. [158]	-
Ethnic minority areas		2021	Guan et al. [159]	-
COVID-19/minority areas		2020	La [129]	Questionnaire
Border areas		2019	Yang et al. [160]	-
Teacher	4	2018	Yan [161]	-
Teacher		2016	Ling [162]	-
Teacher		2013	Zhang [163]	-
Teacher		2011	Fang [164]	-
New generation of migrant workers/Youth	2	2013	Liu [165]	-
New generation of migrant workers/Youth		2013	Hu [166]	-
Cadres	1	2017	Yan [128]	Questionnaire
Young medical workers	1	2008	Bi [130]	Questionnaire and interview
Soldier	1	2000	Feng et al. [127]	Questionnaire
Laid-off workers	1	1998	Ruan [167]	-

**Table 5:** Literature statistics of clusters on social mentality and health research in the WOS before 1 April 2022 (generated by the authors).

Author	Year	Method	Keywords	Content
Zhao et al. [34]	2022	Questionnaire	pandemic; university students; social mentality; mental health; longitudinal study	Changes in social mentality of college students during COVID-19
Santos et al. [168]	2022	Comparative study/self-report scales	compassion; compassionate mind training; care-giving social mentality; caregivers; residential youth care; cluster randomized trial	The social mentality of the caregiver
Xi et al. [169]	2021	Questionnaire	COVID-19 pandemic; Chinese sense of well-being; peace of mind; social support; cross-lag model	Social mentality and happiness
Kou et al. [170]	2021	Participatory action research methods	community gardening; PAR; COVID-19 pandemic; mental health; community building	Community gardening and mental health under COVID-19
Nie et al. [171]	2021	Questionnaire	COVID-19; panic; pandemic-related knowledge; self-efficacy; risk; objective social support	Social mentality under COVID-19
Wang et al. [4]	2020	Questionnaire	COVID-19; mental health; pandemic knowledge; self-efficacy; risk level; family-based social support	Social mentality and mental health under COVID-19
Zhang et al. [172]	2015	Likert scale/Simple material value scale	occupation; cognitive basement of social mentality; ‘smart-selfishness’; creativity; prosocial tendencies; altruism	Social mentality and occupational mental health

## Data Availability

Publicly available datasets were analyzed in this study. These data can be found here: https://login.webofknowledge.com/ (accessed on 1 April 2022), and https://www.cnki.net/ (accessed on 1 April 2022).
